# Recalled Childhood Gender-Related Play Behaviour and Current Gender-Related Occupational Interests in University Students: Examining the Mediating Roles of Gender Compatibility, Goal Endorsement, and Occupational Stereotype Flexibility

**DOI:** 10.3389/fpsyg.2022.927998

**Published:** 2022-07-06

**Authors:** Karson T. F. Kung

**Affiliations:** Department of Psychology, University of Hong Kong, Pokfulam, Hong Kong SAR, China

**Keywords:** developmental, occupation, play, sex, gender

## Abstract

Substantial average gender differences in childhood play behaviour and occupational interests have been well-documented. Recent research shows that childhood gender-related play behaviour longitudinally predicts gender-related occupational interests in adolescence (Kung, 2021). The first aim of the present study was to extend this recent finding by examining whether university students’ recalled childhood gender-related play behaviour predicts their current gender-related occupational interests. The second aim of the present study was to investigate whether gender-related socio-cognitive processes mediate the relation between childhood play behaviour and subsequent occupational interests. University students (260 men, 542 women) completed scales assessing recalled childhood gender-related play behaviour, gender-related occupational interests, gender typicality, gender contentedness, agentic goal endorsement, communal goal endorsement, and gender-related occupational stereotype flexibility. In the present study, recalled childhood gender-related play behaviour predicted gender-related occupational interests in both men and women. In men, gender typicality and gender contentedness mediated the play-interests link. In women, gender typicality and communal goal endorsement mediated the play-interests link. The present study provides further evidence that childhood gender-related play behaviour is related to subsequent gender-related occupational interests. Although the current study has a correlational design, one interpretation of the current findings is that childhood play may influence socio-cognitive processes, such as gender compatibility and goal endorsement, which may in turn shape occupational interests.

## Introduction

### Gender, Childhood Play Behaviour, and Occupational Interests

Average gender differences in children’s play behaviour have been well-documented. By the end of the first year of life, gender differences in toy preferences emerge (e.g., dolls vs. toy cars) (Alexander et al., 2009; Jadva et al., 2010; Lamminmäki et al., 2012; Boe and Woods, 2018; Lauer et al., 2018). By 2–3 years old, boys and girls start to exhibit different play styles (e.g., rough-and-tumble vs. verbal and nurturant) and prefer playmates of their own gender group (e.g., engaging in gender-segregated play) (Maccoby, 1998). Meta-analytic research and large-scale population studies have shown that gender differences in childhood play behaviour are substantial and grow even larger with age (Golombok and Rust, 1993a,b; Golombok et al., 2008; Davis and Hines, 2020). These gender differences have been observed across different continents in Western as well as Asian societies (Yu et al., 2010; Wong and Yeung, 2019; Davis and Hines, 2020).

Another domain that shows consistent and substantial average gender differences is occupational interests. Men tend to be more interested in things-oriented occupations such as mechanics and engineers, whereas women tend to be more interested in people-oriented occupations such as teachers and nurses (Lippa, 1998; Su and Rounds, 2015). Substantial differences between men and women in occupational interests have been reported in meta-analytic research (Su et al., 2009) as well as large-scale cross-cultural research including participants from over 50 nations across the globe (Lippa, 2010a).

Considering that children spend thousands of hours in their early years on play through which they learn and socialise, it has been proposed that childhood gender-related play may lay the foundation for aspects of gender development such as occupational outcomes (Weisgram and Dinella, 2018; Kung, 2022). Qualitative differences in male- and female-typical play may shape differential characteristics associated with male- and female-typical occupations. Male-typical play tends to be risky, competitive, and involve construction, whereas female-typical play tends to focus on nurturance, beauty, and domestic skills (Blakemore and Centers, 2005). Similarly, male-typical occupations tend to be risky, flashy, things-oriented, and realistic, whereas female-typical jobs tend to focus on children, fashion, and helping (Lippa, 2005). Therefore, it is possible that childhood gender-related play behaviour contributes to the development of gender-related occupational interests. Evidence for the play-interests link comes from a recent longitudinal study showing that childhood gender-related play behaviour predicts gender-related occupational interests in adolescence (Kung, 2021). Nonetheless, it remains unknown whether childhood gender-related play behaviour predicts gender-related occupational interests in adulthood. Also, there is limited research on the mechanisms underlying the play-interests link.

It is important to note that, like other aspects of human development, gender development is shaped by various types of influences and their interactions (Hines, 2015; Kung, 2022). Some prior research studies examining either play behaviour or occupational interests have been conducted within theoretical frameworks concerning evolutionary, genetic, and early hormonal influences (e.g., Iervolino et al., 2005; Pellegrini, 2006; Lippa, 2010a; Nicolaou and Shane, 2010; Spencer et al., 2021). The present study focuses on socio-cognitive processes that may explain how childhood play behaviour contributes to subsequent occupational interests. A greater understanding of the play-interests link and its related socio-cognitive processes may facilitate future discussions on how to nurture more diverse occupational interests amongst young people.

### Socio-Cognitive Mechanisms Underlying the Play-Interests Link

Potential socio-cognitive factors include gender compatibility, goal endorsement, and occupational stereotype flexibility. Two key aspects of gender compatibility are gender typicality (perceived similarity between oneself and same-gender peers) and gender contentedness (satisfaction with one’s own gender). Research has shown that children who engage more frequently in play behaviour that is typical of their own gender report higher levels of gender-typicality and gender contentedness; for instance, girls engaging in more female-typical/less male-typical play behaviour show higher levels of gender-typicality and gender contentedness (Egan and Perry, 2001). In addition, studies on adolescents and young adults have found that gender typicality and gender contentedness can predict gender-related occupational interests; for instance, men reporting higher levels of gender typicality and gender contentedness have more male-typical/less female typical occupational interests (Leaper and Van, 2008; Dinella et al., 2014; Koul et al., 2017).

Regarding goal endorsement, little is known about the relation between play and goal endorsement. However, starting from middle childhood, compared with girls, boys endorse agentic goals more (e.g., status, competence) and communal goals less (e.g., altruism, connecting with others) (Block et al., 2018). In adults, agentic and communal goal endorsement predict gender-related occupational interests; for instance, individuals with higher levels of agentic goal endorsement and lower levels of communal goal endorsement have more male-typical/less female-typical occupational interests (Diekman et al., 2010).

Regarding occupational stereotype flexibility, both children and adults exhibit some degree of rigidity when evaluating whether men or women should do certain jobs (i.e., thinking that certain occupations are only for men and certain occupations are only for women) (Liben and Bigler, 2002). Even though the link between stereotypes and gender differences has been widely discussed in the psychological literature (e.g., Fiske, 2010; Master, 2021), there is limited research examining the relations amongst gender-related play, stereotype flexibility, and occupational interests. It is possible that high levels of play behaviour that is typical of one’s own gender may reduce one’s stereotype flexibility which may in turn limit one’s occupational interests. Taken together, different socio-cognitive factors may explain the relation between childhood play behaviour and subsequent occupational interests. Nonetheless, there is a lack of research examining the links amongst these constructs of interest.

### Present Study

Because there are no existing secondary longitudinal data that can be used to address the research gaps, the present study collected primary data using a retrospective recall measure to assess childhood play behaviour. An online survey consisting of a set of self-reported scales was employed. University students took part in the study. The first aim of the present study was to investigate whether recalled childhood gender-related play behaviour predicts current gender-related occupational interests in adults. The second aim of the present study was to investigate whether gender compatibility, goal endorsement, and occupational stereotype flexibility mediate the relation between recalled childhood play behaviour and subsequent occupational interests.

## Materials and Methods

### Participants and Procedures

University students in Hong Kong (260 men, 542 women), aged 18–28 years (*M*_age_ = 20.92, *SD*_age_ = 2.28), completed a set of scales on the online survey platform Qualtrics. The vast majority of the participants (93%) are Chinese. Of the 802 participants, 694 were undergraduate students and 108 were postgraduate students. Participants were recruited through student societies and forums, as well as university notice boards. The study was described as a research project on play preferences in childhood and behaviour in adulthood; the study’s focuses on gender and occupational interests were not mentioned in the recruitment materials.

The study protocol was approved by a research ethics committee at a university in Hong Kong. After completing the online consent form and providing demographic information, participants completed a set of scales. The order of the scales was randomised and counterbalanced across participants. The scales employed in the present study were originally developed in English. University students in Hong Kong typically understand both Chinese and English, but levels of proficiency in the two languages vary across students. Therefore, both Chinese and English versions of the scales were shown to all participants (i.e., each English item was paired with its Chinese translation).

### Measures

#### Predictor

##### Recalled Childhood Gender-Related Play Behaviour

A modified version of the Preschool Activities Inventory (PSAI; Golombok and Rust, 1993a,b) was employed. The PSAI was originally designed to be a scale for parents to report on their preschool children’s play behaviour. In the present study, all the original items were used, but participants were asked to recall their own play behaviour when they were 2–6 years old. The PSAI has 24 items assessing the frequency of gender-related play behaviour. Half of the items are male-typical (e.g., “playing with trains, cars, or airplanes,” “playing with guns,” “engaging in sports and ball games,” “enjoying rough-and-tumble play”) and the other half female-typical (e.g., “playing house,” “playing with girls,” “playing with dolls,” “pretending to be a female character such as a princess”). Responses are given on a 5-point Likert scale (“never,” “hardly ever,” “sometimes,” “often,” “very often”). In the present study, using the standard scoring formula (Golombok and Rust, 1993a), PSAI scores were calculated by first adding the male-typical items, then subtracting the sum of the female-typical items, and finally transforming to a pseudo-*T* scale. The PSAI is designed to measure gender-related play behaviour as a single, unidimensional trait. This one-factor, unidimensional structure is supported by studies factor analysing the items (Golombok and Rust, 1993a,b; Kung et al., 2018). Based on the standard scoring formula, higher PSAI scores indicate more male-typical/less female-typical play, whereas lower PSAI scores indicate less male-typical/more female-typical play. The PSAI and its standard scoring method have been employed in dozens of studies, including several large-scale population studies (e.g., Golombok and Rust, 1993a,b; Iervolino et al., 2005; Golombok et al., 2008). In the current sample, internal consistency was good (men: α = 0.70; women: α = 0.78).

PSAI Scoring Formula:


Score=⁢48.25+1.1*(sum⁢of⁢male⁢-⁢typical⁢items-sum⁢of⁢female⁢-⁢typical⁢items).

#### Outcome

##### Gender-Related Occupational Interests

A 10-item scale assessing male-typical vs. female-typical occupational preferences (MF-Occ) developed by Lippa (2010a,2020) was employed. There are 5 male-typical items (e.g., “car mechanic,” “electrical engineer,” “inventor”) and 5 female-typical items (e.g., “costume designer,” “social worker,” “school teacher”). Participants were asked to rate how much they were interested in each of the occupations, disregarding any concerns related to skills or income. Responses are given on a 5-point scale (1 = “strongly dislike”; 5 = “strongly like”). Following prior research on occupational interests (e.g., Lippa, 1998; Dinella et al., 2014; Kung, 2021), ipsatisation was applied to correct for an elevation response set (tendency for individuals to show interest in many or few occupations); individuals’ mean scores of all items were subtracted from scores of each item. After ipsatisation, MF-Occ scores were computed by averaging the male-typical items and the reversed female-typical items (Lippa, 2010a,^2020^). The MF-Occ is designed to measure gender-related occupational interests as a single, unidimensional trait, with higher scores indicating more male-typical/less female-typical interests and lower scores indicating less male-typical/more female-typical interests. This one-factor, unidimensional structure is supported by studies factor analysing the relevant items assessing gender-related occupational interests (Lippa, 1998, 2010b). The MF-Occ and its unidimensional scoring procedure have been employed in a range of studies, including a large-scale study with over 200,000 participants across 53 nations (Lippa, 2010a). In the current sample, internal consistency was good (men: α = 0.70; women: α = 0.67).

#### Potential Mediators

##### Gender Compatibility

Egan and Perry (2001) developed scales to assess gender typicality and contentedness in children. Following Tate et al. (2015), the scales were modified and adapted for adults. There are 6 items assessing gender typicality and 6 items assessing gender contentedness. The items are gender-specific, assessing one’s compatibility with their own gender group. For instance, for men, a sample gender typicality item reads “I feel that my personality is like most men’s personalities,” and a sample gender contentedness item reads “I never think it might be more fun to be a woman.” The parallel sample items for women read “I feel that my personality is like most women’s personalities” and “I never think it might be more fun to be a man.” Responses are given on a 7-point rating scale (1 = “strongly disagree”; 7 = “strongly agree”). Mean scores of the items were computed, with higher scores on the two scales indicating higher levels of gender typicality and gender contentedness. In the current sample, internal consistency was good (gender typicality: men: α = 0.89; women: α = 0.90; gender contentedness: men: α = 0.80; women: α = 0.72).

##### Goal Endorsement

Items developed by Diekman et al. (2010) were used. There are 14 items assessing agentic goal endorsement (e.g., “power,” “recognition,” “achievement,” “independence,” “competition”) and 10 items assessing communal goal endorsement (e.g., “helping others,” “connecting with others,” “serving humanity,” “spirituality,” “intimacy”). Responses are given on a 7-point rating scale (1 = “not at all important”; 7 = “extremely important”). Mean scores of the items were computed, with higher scores on the two scales indicating higher levels of agentic goal endorsement and communal goal endorsement. In the current sample, internal consistency was good (agentic goal: men: α = 0.88; women: α = 0.87; communal goal: men: α = 0.86; women: α = 0.85).

##### Gender-Related Occupational Stereotype Flexibility

The occupations subscale of the Occupations, Activities, and Traits–Attitude Measure (OAT-AM; Liben and Bigler, 2002) was employed. The measure consists of a list of 20 occupations (e.g., “refrigerator salesperson,” “librarian,” “police officer,” “ballet dancer,” “umpire,” “dietician”). Participants were asked to evaluate the occupations by choosing who should be doing these jobs. There are 5 response options (“only men,” “mostly men, some women,” “both men and women,” “mostly women, some men,” or “only women”). Following Liben and Bigler (2002), the measure was scored as the proportion of “both men and women” responses, with higher scores indicating greater flexibility in gender-related occupational stereotypes. In the current sample, internal consistency was good (men: α = 0.90; women: α = 0.90).

### Analytical Approach

Considering that the key measures are expected to show substantial gender differences, within-gender analyses were conducted to examine relations between the measures. When two variables show substantial gender differences, any relations or bi-variate effects would likely be drastically inflated by the gender differences in the overall sample. For example, both the predictor (childhood play) and the outcome (occupational interests) variables are expected to show gender differences in the same direction. A correlation may emerge in the overall sample due to the gender differences, even if there is no meaningful association within each gender. Moreover, the direction of an effect may differ for men and women. For example, higher scores on the play measure indicate more male-typical/less female-typical play behaviour. As a result, higher scores on the play measure may positively predict gender typicality and contentedness in men but negatively predict gender typicality and contentedness in women. These opposing effects may be offset in the overall sample. These issues regarding inflated effects and opposing effects may not be easily corrected by controlling for gender or interaction terms involving gender. Hence, it is essential to examine the relations amongst the predictor, potential mediators, and outcome using within-gender analyses.

## Results

### Preliminary Analyses and Gender Differences

Scores on the measures were normally distributed (skewness = ± 1) and contained no outliers (*z* = ± 3.29). Independent samples *t*-tests revealed significant gender differences in childhood play behaviour, occupational interests, as well as gender contentedness and gender-related occupational stereotype flexibility. Compared with women, men showed significantly more male-typical/less female typical childhood play behaviour and occupational interests. Men also showed significantly higher levels of gender contentedness but lower levels of occupational stereotype flexibility than did women. Descriptive statistics, inferential statistics, and effect sizes for gender differences are summarised in Table 1.

**TABLE 1:** Gender differences in scores on the measures.

	Men *n* = 260	Women *n* = 542	*t*	Cohen’s *d*
	
	*M* (*SD*)	*M* (*SD*)		
Childhood play behaviour^a^	57.30 (9.63)	33.94 (11.13)	29.02***	2.19
Occupational interests^a^	0.06 (0.52)	–0.44 (0.49)	13.18***	0.99
Gender typicality	4.20 (1.19)	4.17 (1.22)	0.33	0.03
Gender contentedness	4.55 (1.11)	3.66 (1.08)	10.80***	0.81
Agentic goal endorsement	5.28 (0.77)	5.27 (0.69)	0.43	0.00
Communal goal endorsement	5.34 (0.82)	5.45 (0.74)	–1.89	–0.14
Occupational stereotype flexibility	0.55 (0.26)	0.61 (0.25)	–3.45**	–0.26

Independent samples t-tests were conducted. ^a^Higher scores on these measures indicate higher male-typical/lower female-typical traits. Positive Cohen’s ds indicate higher scores in males. *p < 0.05, **p < 0.01, ***p < 0.001.

### Correlations Between Measures

Pearson’s correlations were conducted within each gender to examine relations between scores on the measures. Childhood play behaviour significantly correlated with occupational interests in men, *r*(258) = 0.19, *p* = 0.002, and in women, *r*(540) = 0.38, *p* < 0.001; individuals with more male-typical/less female typical childhood play behaviour showed significantly more male-typical/less female typical occupational interests.

After establishing there was a significant association between the predictor and the outcome, further correlational analyses were conducted to decide which of the remaining variables to be included in subsequent mediation analyses. Variables that correlated significantly with both the predictor and the outcome were subsequently included in mediation analyses. Within-gender correlations are shown in Table 2.

**TABLE 2:** Correlations between potential mediators and predictor or outcome.

	Men (*n* = 260)		Women (*n* = 542)	
	Childhood play behaviour^a^	Occupational interests^a^	Childhood play behaviour^a^	Occupational interests^a^
Gender typicality	0.34***	0.34***	–0.35***	–0.36***
Gender contentedness	0.24***	0.29***	–0.26***	–0.23***
Agentic goal endorsement	0.10	0.06	–0.10*	0.06
Communal goal endorsement	0.12	–0.06	–0.20***	–0.31***
Occupational stereotype flexibility	–0.11	–0.05	0.21***	0.15**

Pearson’s correlations were conducted. ^a^Higher scores on these measures indicate higher male-typical/lower female-typical traits. *p < 0.05, **p < 0.01, ***p < 0.001.

In men, there were significant correlations amongst childhood play behaviour, gender typicality, gender contentedness, and occupational interests in the expected directions. Hence, gender typicality and gender contentedness were used as mediators in the multiple mediator model in men. In women, there were significant correlations amongst childhood play behaviour, gender typicality, gender contentedness, communal goal endorsement, occupational stereotype flexibility, and occupational interests in the expected directions. Hence, gender typicality, gender contentedness, communal goal endorsement, and occupational stereotype flexibility were used as mediators in the multiple mediator model in women.

### Mediation Analyses

Mediation models were tested using bias-corrected bootstrapping analyses with 10,000 resamples (PROCESS macro for SPSS; Hayes, 2013). In these analyses, confidence intervals not including zero indicate significant indirect effects (i.e., significant mediating effects). Multiple mediator models were conducted, with childhood play behaviour entered as the predictor variable and occupational interests entered as the outcome variable.

**FIGURE 1:**
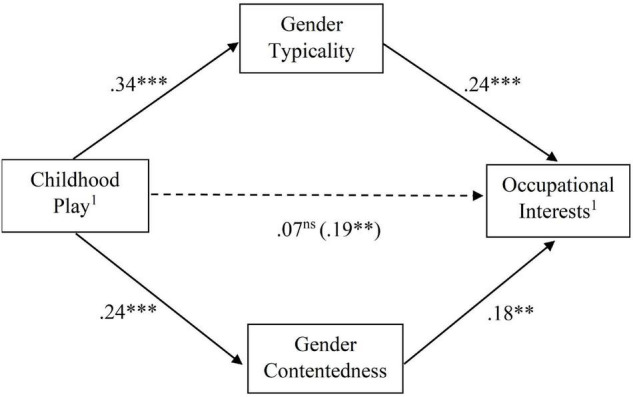
Final multiple mediator model in men. ^1^Higher scores on these measures indicate higher male-typical/lower female-typical traits. Standardised coefficients are reported. For the path from gender-related childhood play to gender-related occupational interests, the coefficient within the brackets indicates the total effect (without considering the mediators) and the coefficient outside the brackets indicates the direct effect (after taking into account the mediators). In this model, the relation between childhood play and occupational interests became non-significant after the mediators were introduced, indicating full mediation. *p < 0.05, ^**^p < 0.01, ^***^p < 0.001.

In men, both mediators were significant (gender typicality: β = 0.08, *SE* = 0.03, 95% CI = [0.03, 0.14]; gender contentedness: β = 0.04, *SE* = 0.02, 95% CI = [0.01, 0.09]). This multiple mediator model in men is depicted in Figure 1. The path from childhood play to occupational interests became non-significant after the mediators were introduced (see Figure 1), which may be interpreted as full mediation.

**FIGURE 2:**
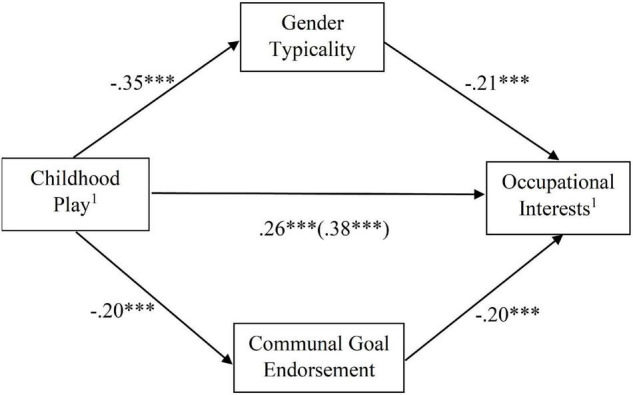
Final multiple mediator model in women. ^1^Higher scores on these measures indicate higher male-typical/lower female-typical traits. Standardised coefficients are reported. For the path from gender-related childhood play to gender-related occupational interests, the coefficient within the brackets indicates the total effect (without considering the mediators) and the coefficient outside the brackets indicates the direct effect (after taking into account the mediators). In this model, the relation between childhood play and occupational interests became weaker after the mediators were introduced, indicating partial mediation. *p < 0.05, ^**^p < 0.01, ^***^p < 0.001.

In women, in a multiple mediator model, gender typicality and communal goal endorsement were significant mediators (gender typicality: β = 0.07, *SE* = 0.02, 95% CI = [0.04, 0.10]; communal goal endorsement: β = 0.04, *SE* = 0.01, 95% CI = [0.02, 0.07]), but gender contentedness and occupational stereotype flexibility were not significant mediators (gender contentedness: β = 0.01, *SE* = 0.01, 95% CI = [–0.01, 0.04]; occupational stereotype flexibility: β = 0.01, *SE* = 0.01, 95% CI = [–0.01, 0.02]). For clarity of presentation, the final multiple mediator model included only gender typicality and communal goal endorsement (gender typicality: β = 0.07, *SE* = 0.02, 95% CI = [0.04, 0.11]; communal goal endorsement: β = 0.04, *SE* = 0.01, 95% CI = [0.02, 0.07]). This final multiple mediator model in women is depicted in Figure 2. The path from childhood play to occupational interests became weaker but remained significant after the mediators were introduced (see Figure 2), which may be interpreted as partial mediation.

### Additional Analyses

In a set of additional multiple mediator models, demographic variables (age, degree level, ethnicity) were included as covariates to see if taking into account these control variables would change the results. The inclusion of these variables did not change the results, and the models yielded highly similar findings.

Finally, to provide further justification for the within-gender analysis approach, bivariate correlations in the overall sample were examined. In the overall sample, occupational stereotype flexibility did not significantly correlate with childhood play behaviour, *r*(800) = 0.00, *p* = 0.85, or occupational interests, *r*(800) = 0.02, *p* = 0.51, suggesting that occupational stereotype flexibility cannot be included as a mediator in a mediation model. By contrast, as shown previously in within-gender analyses, in women, occupational stereotype flexibility correlated significantly with both the predictor and the outcome in the expected direction, meeting the criteria to be included as a mediator. In addition, in the overall sample, lower levels of gender typicality correlated significantly with more male-typical/less female-typical childhood play, *r*(800) = –0.10, *p* = 0.005, and occupational interests, *r*(800) = –0.11, *p* = 0.002, suggesting that gender typicality can be included as a mediator for women but not for men. By contrast, as shown previously in within-gender analyses, gender typicality correlated significantly with both the predictor and the outcome in the expected directions separately in men and in women. The above examples demonstrate that statistical testing based on the entire sample would likely lead to a series of problematic decisions in the process of conducting mediation analyses. In theory, it would be possible to consider interactions with gender in every single step. However, these interaction terms or moderation analyses may not effectively capture and adjust the inflated/opposing effects. Also, consistently examining interactions with gender may generate a large number of redundant analyses based on the overall sample, because any significant interactions would eventually lend support for the use of within-gender analyses. Therefore, the current approach of conducting within-gender analyses from the beginning is necessary and relatively concise.

## Discussion

### Overview and Interpretation of Findings

The present study found that recalled childhood gender-related play behaviour was a significant predictor of current gender-related occupational interests in university students. This finding extends a recent longitudinal study reporting a link between childhood gender-related play behaviour and adolescent occupational interests (Kung, 2021). The present study also found that gender compatibility and goal endorsement may explain the play-interests link, although the relevant socio-cognitive processes may differ for men and women.

In the present study, in multiple mediator models, whilst gender typicality was a significant mediator in both men and women, gender contentedness was a significant mediator in men only. The current findings regarding gender typicality and contentedness are broadly in line with previous research findings. Although both aspects of gender compatibility have been found to be predictive of occupational interests, gender typicality has been a more reliable and robust predictor across studies compared with gender contentedness (Leaper and Van, 2008; Dinella et al., 2014; Koul et al., 2017).

Regarding goal endorsement, agentic goal endorsement was generally not related to either play behaviour or occupational interests, but communal goal endorsement was a significant mediator in women. The present study did not find any significant or apparent gender differences in either agentic or communal goal endorsement. Whilst prior research has produced mixed findings regarding gender differences in agentic goal endorsement, significant gender differences in communal goal endorsement have been more consistently detected across studies (Diekman et al., 2010; Block et al., 2018). The lack of significant gender differences in goal endorsement in the present study may have undermined the link of goal endorsement to childhood play behaviour and to occupational interests.

Regarding gender-related occupational stereotype flexibility, the mediation approach produced non-significant results. However, in women, most of the bivariate correlations between the different mediators and the predictor/outcome were significant and in the expected directions, including those involving occupational stereotype flexibility. It is noteworthy that the present study did not have an enormous sample and there were twice as many women as men in the current sample. The mediating effects of the socio-cognitive factors may be more salient and significant in a larger sample of men and women. Taken together, the current findings add to the literature by showing that childhood play behaviour may influence subsequent occupational interests through certain socio-cognitive processes and that these processes may be particularly important in girls’ and women’s development.

### Implications and Limitations

The present study suggests that gender differences in occupational outcomes may be rooted in early childhood. One implication of the current findings is that parents and educators may be able to reduce gender differences in occupational interests through encouraging more diverse play preferences amongst young children. Researchers have proposed various approaches to diversifying play behaviour in boys and girls (Weisgram and Dinella, 2018) and have reported attempts of using gender-typed colours (e.g., pink vs. blue) to change children’s play preferences (Weisgram et al., 2014; Wong and Hines, 2015). However, further research is needed to systematically test and document the effectiveness of different approaches in changing children’s play behaviour and its related socio-cognitive processes. Further research is also needed to investigate the malleability of the relevant socio-cognitive processes and whether any changes in these processes may affect occupational interests in young people.

A limitation of the present study is that a retrospective recall measure was used to assess childhood gender-related play behaviour. This measure may suffer from recall bias. Nonetheless, more than 60 retrospective research studies asking adults to recall their childhood gender role behaviour, including play, consistently found that gay men and lesbian women recalled more gender non-conforming play than did heterosexual adults (Bailey and Zucker, 1995; Zucker, 2008). These findings were subsequently confirmed by a longitudinal population study showing that, compared with heterosexual individuals, non-heterosexual individuals exhibited more gender non-conforming play in childhood (Li et al., 2017). These retrospective and longitudinal findings suggest that the recall method can offer some degree of reliability. However, further longitudinal research is needed to assess specifically the reliability of adults’ recall of childhood play behaviour.

Another limitation is that the present study is correlational. Statistically, the correlational design limits conclusions regarding causality and directionality. Noteworthy is that it would be very challenging, if not impossible, to manipulate all the predictor and mediators at the same time to establish a causal chain. Due to problems with manipulations and potential statistical confounds, proving causal mediation is hard, especially when different developmental stages and multiple socio-cognitive mediators are involved. In practice, mediation analyses are often conducted and interpreted based on chronological sequence and theoretical frameworks. In the current study, childhood play experiences preceded other constructs of interest, and the theoretical frameworks employed often treat occupational interests as the outcome. Therefore, despite the correlational design of the current study, the analytical approach and the interpretation of findings in the study are adequate both temporally and theoretically. Further longitudinal and experimental research focusing on specific paths is needed to corroborate the current findings and to establish the directions of the effects.

## Conclusion

The present study suggests that childhood gender-related play behaviour may be an important factor in the development of gender-related occupational interests. The correlational design of the current study means that no conclusions can be made regarding causality, but it is possible that play behaviour may exert influences on gender-related occupational interests through aspects of gender compatibility and goal endorsement. Socio-cognitive processes appear to be more important in explaining the play-interests link in women, but the relevant relations may become more salient in a larger sample of men. These findings highlight the possibility that diversifying play behaviour in boys and girls may alter the related socio-cognitive processes, which may in turn reduce gender gaps in occupational outcomes. Nonetheless, because of the correlational nature of the current findings, further research employing different designs and methods is needed to corroborate and extend the current findings.

## Data Availability Statement

The datasets presented in this article are not readily available because of ethical and administrative policies. Requests to access the datasets should be directed to corresponding author.

## Ethics Statement

The studies involving human participants were reviewed and approved by the Departmental Research Ethics Committee, Department of Psychology, University of Hong Kong. The patients/participants provided their written informed consent to participate in this study.

## Author Contributions

KTFK designed the study, collected and analysed the data, as well as wrote and approved the manuscript.

## Conflict of Interest

The author declares that the research was conducted in the absence of any commercial or financial relationships that could be construed as a potential conflict of interest.

## Publisher’s Note

All claims expressed in this article are solely those of the authors and do not necessarily represent those of their affiliated organizations, or those of the publisher, the editors and the reviewers. Any product that may be evaluated in this article, or claim that may be made by its manufacturer, is not guaranteed or endorsed by the publisher.
